# Climate Change Challenge Faced by Italian Children: A Nationwide Study

**DOI:** 10.3390/healthcare12171797

**Published:** 2024-09-09

**Authors:** Livio Provenzi, Michelle A. Ovalles Gomez, Simona Frassone, Cinzia Pilo, Elisa Angiolini, Serena Barello

**Affiliations:** 1 Department of Brain and Behavioral Sciences, University of Pavia, Viale Golgi 19, 27100 Pavia, Italy; livio.provenzi@unipv.it (L.P.); michellealexan.ovallesgomez01@universitadipavia.it (M.A.O.G.); 2 Developmental Psychobiology Laboratory, IRCCS Mondino Foundation, Via Mondino, 2, 27100 Pavia, Italy; 3 ScuolAttiva Onlus, Via del Corso, 300, 00186 Roma, Italy; s.frassone@scuolattiva.it; 4 Triplepact SRL, Via Pier Paolo Pasolini, 4, 20251 Milano, Italy; cinzia.pilo@fondazionereb.com; 5 Institute of Psychology and Education, University of Neuchâtel, Espace Tilo-Frey 1, 2000 Neuchâtel, Switzerland; elisa.angiolini@unine.ch; 6 Behavioral Health Psychology Laboratory, University of Pavia, Piazza Botta 11, 27100 Pavia, Italy

**Keywords:** eco-anxiety, children, climate change, environment, survey

## Abstract

Climate change threatens environmental stability and human health, with limited research on younger children’s perceptions. This study examines Italian primary school children’s views on climate change to guide educational and policy strategies. Surveying 973 children (5–11 years) from different regions, findings showed high awareness (93%) and concern (63%) about climate change. Regional differences indicated greater concern in the south. Gender disparities revealed females were more concerned and experienced more sleep difficulties. Younger children had stronger emotional responses, while older ones showed increased pro-environmental behaviors. Despite moderate self-confidence in effecting change, a strong sense of responsibility and trust in adults was prevalent. These results highlight the need for early, continuous climate education tailored to regional, age, and gender-specific needs. Addressing children’s views on climate change can help educators and policymakers foster resilience and proactive attitudes, supporting the development of informed and engaged future stewards of the planet.

## 1. Introduction

Climate change poses formidable challenges to planetary health, threatening environmental stability and human health. While significant attention has been given to adolescents’ perspectives on climate change [1], younger children’s perceptions and reactions to these challenges remain less known. School-age children are the future stewards of our planet and are particularly vulnerable to environmental impacts; therefore, understanding their awareness, concerns, and coping strategies is essential. By understanding how children perceive climate change, educators can design both informative and engaging curricula, promoting long-term environmental stewardship.

As children’s awareness of climate change is influenced by multiple factors, including education and regional conditions [2], educational and informational interventions are key in shaping their perceptions of environmental issues. Addressing children’s views on and engagement with climate change challenges can inform effective environmental education programs, fostering resilience and proactive attitudes towards climate challenges [3]. Studies have indicated that climate change can profoundly impact young people psychologically, leading to anxiety, stress, and helplessness [4]. Involving children’s voices in environmental discourse empowers them to contribute meaningfully to future sustainability efforts [5,6], thus informing and engaging a generation in addressing planetary health challenges.

In Italy, a country with diverse climates and increasingly frequent extreme environmental events, discovering children’s perspectives on climate change is specifically relevant. Individual and regional disparities in climate education and exposure to climate impacts can lead to significant differences in children’s awareness, concerns, and coping mechanisms. Understanding such variations is essential for developing targeted strategies that address the specific needs and challenges faced by children across Italy. This study aims to explore the attitudes and perspectives of a large cohort of Italian children towards climate change and planetary health. By examining the levels of awareness, perceived relevance of climate change, concerns about the future, and coping mechanisms, we seek to provide a comprehensive understanding of how younger populations are affected by and respond to environmental stressors. The findings will inform country-based educational, policy, and mental health strategies, thereby supporting children in navigating the complexities of a changing planet while fostering their contributions to a sustainable future.

## 2. Materials and Methods

### 2.1. Sample and Procedures

This study was part of a larger educational national project entitled A Scuola di Acqua, Sete di Futuro (English translation, *Learning About Water, Thirst for the Future*) conducted with the collaboration of ScuolAttiva Onlus. The project aimed to raise awareness among young people on topics related to hydration and environmental sustainability. Data were obtained through an online survey administered between 8 April 2024 and 18 May 2024 to a large sample of children attending Italian primary schools and participating in the educational project, recruited through the ScuolAttiva mailing list. All children who participated in the educational project also filled out the survey. The fully anonymized database was received by the researchers, who then analyzed the data.

The survey included a series of questions to profile the sample from a socio-demographic perspective (i.e., gender, age, place of residence, primary school grade, and recent exposure to extreme climatic events). Further questions were designed to explore four main thematic clusters: (1) engagement with the climate change challenge (Table 1), (2) concerns about the climate change challenge (Table 2), (3) sense of agency towards the climate change challenge (Table 3), and (4) hopefulness towards the climate change challenge (Table 4). The study adhered to the principles outlined in the Declaration of Helsinki and received approval from the independent ethics committee of Pavia (protocol n. 0042065-24, 31 July 2024). Informed consent for the children to participate in the project and the related survey was obtained from their parents.

### 2.2. Plan of Analysis

According to the explorative nature of the present study, descriptive statistics were obtained for all the survey items. The distribution of the responses to each item was contrasted by gender (i.e., females vs. males), place of residence (i.e., north, center, south), grade (i.e., from first to fifth primary school grades), and exposure (i.e., no vs. yes) by means of separate chi-squared tests. The Bonferroni correction was adopted to address the multiple comparison bias. Significant differences were detected at *p* < 0.01. Statistical analyses were performed with Jamovi software for Windows 11, version 2.4.11.020.

## 3. Results

**Figure 1:**
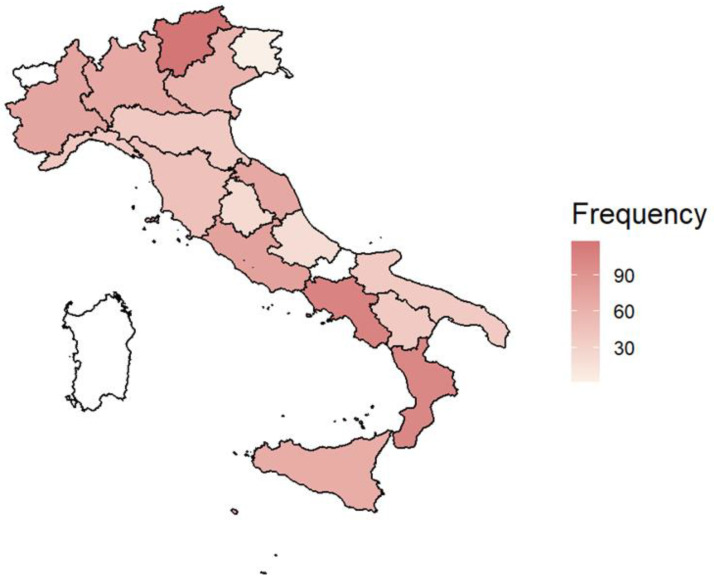
Geographical distribution of survey respondents.

A cohort of 973 children (469 females, 48%) was enrolled across the five primary school grades (i.e., first grade, n = 159, 16%; second grade, n = 126, 13%; third grade, n = 268, 28%; fourth grade, n = 219, 23%; and fifth grade, n = 201, 21%) throughout Italy. The age range was 5 to 11 years (mean age = 8.53 years, standard deviation = 1.41). The geographical distribution is reported in Figure 1. Among respondents, 417 (43%) came from regions recently exposed to extreme environmental and climate change events (e.g., flooding, seismic events, wildfires, drought). The survey items are reported in Table 1, Table 2, Table 3 and Table 4.

**Figure 2:**
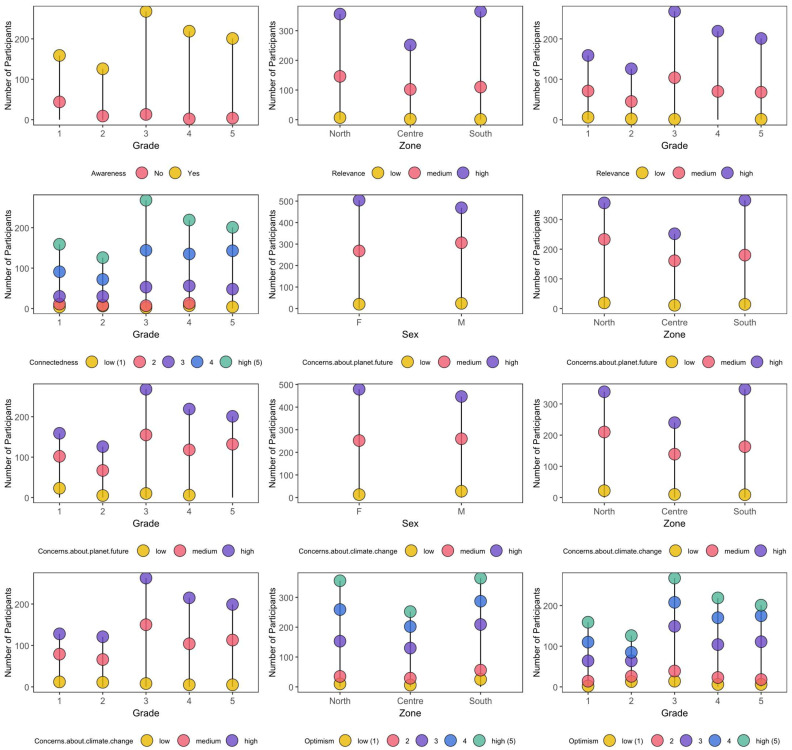
Significant variations in children’s perspective on climate change challenges.

The main findings are graphically summarized in Figure 2. Children’s awareness was very high across the entire sample: 901 children (93%) have heard about climate change. Awareness steadily increased from 72% in first-grade children to 93% in second-grade children, χ^2^(16) = 14.66, *p* < 0.001. Perceived relevance was reported as high by 615 children (63%). Significant variations were detected by zones: 255 children (70%) from the south reported climate change challenges to be very relevant, a higher percentage when compared to counterparts from the north (59%) and the center (60%), χ^2^(4) = 15.01, *p* = 0.005. Moreover, the percentage of children rating climate change as an irrelevant topic decreased from 4% among first-grade students to 0% among third-to-fifth-grade children, χ^2^(8) = 21.81, *p* = 0.005. Within the tested sample, 40% of children (n = 388) reported feeling very connected to climate change challenges. Connectedness was higher in younger children (43 to 46% for those in first to third grades) compared to older ones (29% for those in fifth grade), χ^2^(16) = 37.55, *p* = 0.002. Only 44 children (5%) reported not being concerned whatsoever about the planet’s future. This percentage was higher in those enrolled in the first grade (n = 115, 72%), χ^2^(8) = 57.98, *p* < 0.001. Among females, the percentage of children highly concerned was greater than among males, respectively, 47% vs. 35%, χ^2^(2) = 14.66, *p* < 0.001. As for zones, 185 children (51%) from the south reported being highly concerned about the planet’s future, a percentage that was lower among children from the north (35%) and the center (36%), χ^2^(4) = 23.02, *p* < 0.001. Less than half of the sample was specifically concerned about climate change, n = 414 (45%). This percentage was greater among females (47%) than males (42%), χ^2^(2) = 8.36, *p* = 0.015, as well as among children from southern Italy (53%) compared to counterparts from the north (38%) and the center (42%), χ^2^(4) = 19.69, *p* < 0.001. While up to 9% of the sample reported not being worried at all, this percentage was significantly lower from third grade onward (between 2 and 3%), χ^2^(8) = 24.42, *p* = 0.002. A relatively large proportion of children (n = 389, 40%) reported sleep difficulties because of recursive thoughts about the climate. This percentage was significantly higher among females, n = 230 (46%), compared to males, n = 159 (34%), χ^2^(1) = 13.94, *p* < 0.001, as well as among children from the south, n = 173 (47%), compared to counterparts from the north, n = 134 (38%) and the center, n = 82 (33%), χ^2^(2) = 14.99, *p* < 0.001. A similar percentage of children (n = 378, 39%) disclosed at least one climate-related nightmare during the last months with no significant differences by gender, zone, and grade.

Most children felt a responsibility to make a significant impact on planetary health (n = 930, 96%) and reported a heightened sense of duty (n = 946, 97%). Children also showed mild (n = 166, 17%), enough (n = 364, 37%), or great (n = 193, 20%) self-confidence, showing no significant variations by gender, zone, and grade. In terms of commitment, children reported making pro-environmental behaviors seldom (n = 629, 65%) or often (n = 325, 33%), whereas the percentage of those disclosing no commitment at all decreased from 3% to 6%, respectively, in first- and second-grade students to 1% among those in third and fifth grade. Only a minor proportion of children reported the lowest score for optimism (n = 41, 4%). Nonetheless, this proportion is higher in the south of Italy (7%) compared to the north (3%) and the center (2%), χ^2^(8) = 24.31, *p* = 0.002. Moreover, 31% of first-grade children and 33% of second-grade children reported the highest score for hopefulness compared to 21% and 26% of third- to fifth-grade students, χ^2^(16) = 37.55, *p* = 0.003.

## 4. Discussion

The present study aimed to explore Italian children’s perspectives on climate change, focusing on awareness, concerns, agency, and hopefulness. The findings reveal that awareness of climate change among Italian children is exceptionally high, indicating that even at a young age, children are not only informed about the issue but also deeply engaged with its implications. However, there are significant differences in how climate change is perceived across different age groups, genders, and geographical regions. The high level of awareness and perceived relevance, particularly among children from the south of Italy, may reflect their direct experiences with extreme environmental events, underscoring the role of local context in shaping environmental awareness [7].

The study reveals a strong sense of responsibility and duty towards environmental protection among children, which is crucial for fostering long-term pro-environmental behaviors [8]. This strong sense of responsibility is a key factor in fostering long-term pro-environmental behaviors, suggesting that children are not only aware of the challenges posed by climate change but also feel personally obligated to act. However, significant concerns about the planet’s future and climate change are evident, particularly among older children and females, suggesting that climate-related anxiety is a critical issue that needs to be addressed [9]. These concerns manifest in heightened levels of climate-related anxiety, which is becoming increasingly recognized as a critical issue that needs to be addressed. The high prevalence of sleep difficulties and nightmares related to climate change underscores the psychological impact of environmental issues on children, highlighting the need for supportive interventions to help them cope with these concerns [4]. This finding highlights the urgent need for supportive interventions to help children cope with their anxieties and develop resilience in the face of environmental uncertainty.

Children’s sense of agency and self-confidence in making a difference was moderate, indicating the importance of empowering children through education and engagement in environmental actions [10]. This suggests that while children were aware of the importance of environmental protection, they might have felt limited in their capacity to effect change. This underscores the importance of empowering children through education and active engagement in environmental actions. By enhancing their sense of agency, children can be better equipped to contribute meaningfully to environmental sustainability efforts. The variation in optimism and hopefulness across different age groups and regions suggests that tailored and multi-stakeholder interventions are needed to foster engagement and a positive outlook towards climate challenges and future sustainability [11,12]. Ensuring that children remain hopeful and engaged is crucial for sustaining long-term environmental action.

### Strengths and Limitations

The present study has specific strengths and limitations. The survey benefited from a large sample size of children attending primary schools across Italy, which supports generalizability of findings to the national context. The study was also able to target children who were already engaged in educational activities regarding climate change challenges. Finally, the study reported on children’s attitudes toward climate change and environmental challenges for the first time in Italy with such a broad focus. At the same time, the survey methodology may have introduced biases related to self-disclosure and perceptions. The data collection was cross-sectional, and the age-related effects do not reflect actual developmental trajectories, but rather differences between children of different ages. The participation in the educational program might have increased children’s closeness to the topics of the survey; future studies should imply a pre-post assessment to evaluate the effectiveness of such educational interventions.

## 5. Conclusions

In conclusion, this study provides valuable insights into Italian children’s perspectives on climate change, emphasizing the need for targeted educational and psychological interventions. These interventions are essential to support children as they navigate the complex environmental challenges they face. The findings highlight the importance of addressing both the cognitive and emotional dimensions of children’s engagement with climate change. Future research should focus on conducting longitudinal studies to track changes in children’s perceptions and coping mechanisms over time. Additionally, further exploration of the effectiveness of various educational approaches in fostering environmental stewardship and resilience among young populations is warranted. By understanding and addressing the diverse needs and perspectives of children, we can better prepare the next generation to meet the challenges of a changing climate.

## Figures and Tables

**Table 1:** Engagement with the climate change challenge.

Item n.	Item Label	Item Text	Rating
1	Awareness	Did you hear about climate change?	Dichotomous (no; yes)
2	Relevance	How relevant is the climate change challenge for you?	Likert scale (3-point)
3	Connectedness	How close do you feel toward the climate change challenge?	Likert scale (5-point)

**Table 2:** Concerns about the climate change challenge.

Item n.	Item Label	Item Text	Rating
4	Concerns about the planet’s future	How much do you feel concerned about the future of our planet?	Likert scale (3-point)
5	Concerns specific to climate change risks	How much do you feel concerned about climate change risks?	Likert scale (3-point)
6	Sleep difficulties	Have you ever had difficulties in sleeping because of thinking about the climate change challenge?	Dichotomous (no; yes)
7	Nightmares	Did you ever have nightmares about the climate change challenge?	Dichotomous (no; yes)

**Table 3:** Sense of agency towards the climate change challenge.

Item n.	Item Label	Item Text	Rating
8	Responsibility	Do you feel responsible for caring for the planet’s health?	Dichotomous (no; yes)
9	Sense of duty	Do you think your contribution is needed to care for the planet’s health?	Dichotomous (no; yes)
10	Self-confidence	How much do you feel you can cope with the climate change challenge?	Likert scale (5-point)
11	Commitment	How often do you engage in pro-environmental behaviors?	Likert scale (3-point)

**Table 4:** Hopefulness towards the climate change challenge.

Item n.	Item Label	Item Text	Rating
12	Optimism	How much healthier do you think the planet will be in 5 years?	Likert scale (5-point)
13	Trust in adults	Do you trust adults to cope with climate change challenges?	Dichotomous (no; yes)

## Data Availability

The data are available upon reasonable request from the corresponding author.
